# Gel card kit to detect recent homologous blood transfusion for anti-doping purpose. A proof of concept and assessment of limitations

**DOI:** 10.3389/fspor.2025.1597235

**Published:** 2025-06-03

**Authors:** Pedro Antônio Castelo Teixeira, Rachel Santos Levy, Tiago Ascenção Barros, Allan de Oliveira de Azevedo, Monica Costa Padilha, Henrique Marcelo Gualberto Pereira

**Affiliations:** ^1^ Brazilian Doping Control Laboratory—LBCD/UFRJ, Rio de Janeiro, Brazil; ^2^ Hemotherapy and Hematology Unit, University Hospital Clementino Fraga Filho, HUCFF/UFRJ, Rio de Janeiro, Brazil

**Keywords:** doping control, blood transfusion, gel card, flow cytometry, red blood cell

## Abstract

Homologous blood transfusion (HBT) is used illicitly by some athletes to increase red blood cell (RBC) mass. The cytofluorometric techinique detects HBT by distinguishing between two different RBC populations. Although effective, this method is costly and time-consuming. Gel card kits (ID-Card, BioRad), which are widely used in clinical practice, offer faster and more cost-effective alternative. This study evaluates the ability of Gel cards to differentiate between two RBC populations, when compared with cytofluorometry. Blood samples from volunteers were phenotyped for minor antigens (C, c, E, e, K, k, Jka, Jkb, Fya, Fyb, S and s) using flow cytometry. Non-compatible blood samples, differing in minor antigens groups, were mixed in different proportions (10%, 5% and 3% of donor blood). Gel cards were able to detect 5% of the minor population when the donor expressed C, c, E and e antigens. The limit of detection for K, Jka and Jkb antigens is 3%. However, Gel cards were not able to detect double RBC population when the donor expressed these antigens. Similarly, Gel cards could not distinguish between two RBC populations differing in Fya, Fyb, S and s expression, as the antigen-positive cells were not concentrated at the top of the Gel but were instead dispersed throughout it. Further studies are required to evaluate the ability of Gel cards to detect other antigens (e.g., M, N, P1, Le^a^, Le^b^, Lu^a^, Lu^b^). Although the Gel card is less sensitive than cytofluorometry, it represents a more cost-effective and practical alternative for detecting recent blood transfusions.

## Introduction

Homologous blood transfusion (HBT) consists of an infusion of blood (either whole blood or blood components) from one individual into another who is ABO- and Rh-compatible. This practice is an effective and immediate method for increasing the number of red blood cells (RBCs) in blood circulation, thereby enhancing and optimizing oxygen delivery to organs and tissues, including skeletal muscle (1, 2). HBT began to gain popularity among athletes in the 1970s, particularly in endurance sports such as long-distance running, cycling, and cross-country skiing, as means of enhancing aerobic capacity by delaying onset of exhaustion (3). Following the commercial introduction of recombinant human erythropoietin (rhEPO) for medical use in the 1980s, the practice of HBT as a doping method appeared to decline. Athletes began using rhEPO to increase the number of circulating RBCs due to its potency, ease of access, and the lower complexity of administration compared to transfusion-based methods (4). However, the development and implementation of direct detection methods for both rhEPO and HBT in the early 2000s likely led athletes to adopt autologous blood transfusion (ABT), a technique that consists of a reinfusion of their own stored blood after several weeks of storage (5). Although the ABT procedure is relatively complex and costly—requiring logistical planning for blood withdrawn, refrigerated storage, and reinfusion into the donor's circulation shortly before the competition—the absence of HBT-positive cases during the 2010s, combined with the lack of a direct detection method for ABT, raised the suspicions that athletes may have shifted toward using ABT as doping strategy (5). However, one HBT-positive case reported in Brazil in 2019, along with two additional cases during the Tokyo 2020 Olympic Games, brought renewed attention to the use of HBT as a doping method again (6).

There are two direct techniques to detect HBT. One is based on forensic DNA analysis, which identifies the presence of two distinct DNA profiles in a blood sample (7, 8). The other consists of RBC analysis by flow cytometry to identify the presence of two distinct RBC populations in a blood sample (9, 10). The DNA-based method is highly specific, as it analyzes between 16 and 24 short tandem repeats (STR)—small repeated sequences of nucleotides located at different positions in the non-coding region of human DNA—thus minimizing the risk of false negative results. Despite its high specificity and low limit of detection (LOD), estimated at approximately 2% of donor blood *in vitro*, no trace of donor DNA was detected even one day after the transfusion of 150 ml of RBC concentrate in *in vivo* studies (11). RBCs are enucleated cells and therefore do not contain DNA. The source of DNA in the blood originates from nucleated white blood cells (WBCs). These WBCs can be efficiently removed from blood samples by using leukodepletion filters during the preparation of RBC concentrates, significantly reducing the number of DNA-containing cells and making HBT detection by this method nearly impossible (12). The flow cytometric method for detecting HBT is a robust test based on Fluorescence Assisted Cell Sorter (FACS), which recognizes two phenotypically distinct RBC populations (9). Approximately 300 surface markers on the RBC membrane are recognized by the International Society of Blood Transfusion (ISBT), and each individual expresses a specific pattern of these antigens (13). Considering compatibility in the major blood groups (ABO and Rh systems) between donor and recipient to reduce the risk of hemolytic transfusion reactions or alloimmunization, the HBT detection method focuses on the presence or absence of 12 minor antigens (C, c, E, e, Fya, Fyb, Jka, Jkb, K, k, S, s) (14). Although this assay is highly efficient and sensitive, it involves high costs related to equipment, materials, and reagents. These costs become even more significant when the number of analyses is low compared to other routine tests. The sample preparation for the cytofluorimetric method is time-consuming, as it requires multiple steps including washing and incubating RBCs with primary antibodies that recognize specific antigens, followed by additional washes and incubation with a secondary antibody conjugated to a fluorescent probe. The expression or non-expression of antigens is then analyzed using a flow cytometer. Additionally, operating the equipment requires an expert professional specialized in flow cytometry, which increases the overall cost of the method. The characterization of an HBT event relies on the detection of RBCs that either express or do not express minor antigens, visualized as two distinct peaks in the histogram (one corresponding to the donor and the other to the recipient). The LOD of the cytofluorimetric analysis has been estimated to be below 1% (99% recipient blood mixed with 1% donor blood). Hence, this method is the preferred choice in anti-doping laboratories worldwide, both as Initial Testing Procedure (ITP) and Confirmation Procedure (CP) for detecting HBT (10, 15, 16). According to the World Anti-Doping Agency (WADA) regulations, the use of the HBT detection method is not mandatory in the accredited laboratories. Due to the misconception that HBT is no longer used by athletes—a belief challenged by cases observed during the Tokyo 2020 Olympic Games—the number of HBT analysis requests has decreased worldwide. According to the latest WADA statistics (17), only 483 HBT tests were conducted by the accreditated laboratories worldwide in 2022, with no positive cases reported. In fact, the low number of HBT tests is also assigned to the high costs associated with the method, as previously mentioned. However, there is a clear discrepancy when compared to the total number of Athlete Biological Passport (ABP) tests, which was sixty times higher (31,246 samples). If a portion of these samples were also tested for HBT as part of a targeted strategy, detection efficiency could be improved. Nonetheless, maintaining HBT testing on a routine basis remains an economic challenge. The optimization of costs in anti-doping activities is a major concern, motivating the scientific community to explore alternative methods such as Dried Blod Spot (DBS), which has already been incorporated into anti-doping protocols for the detection of testosterone esters (18). In this context, the use of HBT by athletes may be underestimated, as a consequence of the limited number of WADA-accredited laboratories offering flow cytometry-based analysis. Therefore, alternative methodological approaches that are more cost-effective and financially viable would be beneficial.

The Gel card kit, also known as micro Gel column technique, is a diagnostic tool widely used for blood phenotyping in blood banks and hospital immunohematology laboratories. Gel cards employ a Gel matrix technology to detect and identify major and/or minor blood group antigens with high sensitivity and specificity. This technique is based on the principle of RBC agglutination. The Gel matrix within the card contains antibodies specific to various RBC surface antigens. When a blood sample is applied to the Gel card, RBCs expressing the corresponding antigens will bind to their specific antibodies. This interaction leads to visible agglutination or RBCs clumps, primarily at the top of the Gel but also distributed throughout the Gel matrix. When the RBCs in the sample do not express the corresponding antigen, the cells migrate to the bottom of the Gel (19, 20). These kits are simple, easy to handle, cost-effective, and require only a few minutes to perform. Moreover, the presence of double RBC populations—one at the top (expressing the antigen) and the other at the bottom (non-expressing the antigen) of the Gel matrix—is frequently observed by healthcare professionals in routine hospital practice, reflecting recent blood transfusions received by the patients (21). Thus, this study aimed to evaluate the capability of the Gel card kits to distinguish two RBC populations in a blood sample, differentiated based on a panel of 12 minor antigens, and to compare the performance of the Gel card kit with the cytofluorimetric method currently used for HBT detection in antidoping applications. The feasibility of implementing the Gel card approach as an ITP in anti-doping analysis was evaluated.

## Experimental

### Blood samples

Approval for the study was obtained from the ethics committee of Federal University of Rio de Janeiro (84097424.4.0000.5257). Twenty-two (22) healthy, non-athlete volunteers, of both genders (13 males and 9 females), aged between 24 and 50 years, representing all the major blood groups (A, B, AB, O), had peripheral whole blood collected in EDTA tubes (K2EDTA, BD Vacutainer®). Each blood sample was phenotyped for 12 minor antigens (C, c, E, e, K, k, Jka, Jkb, Fya, Fyb, S, s) using flow cytometry (Table 1). To prevent interference in the Gel card assay due to pre-existing agglutination, sample mixtures were prepared between whole bloods from volunteers with matching ABO blood system. Bloods from individuals 2, 3, 4, 6, 7, 12, 13 and 15 were mixed based on incompatibility in their minor antigens. These mixtures were prepared in different proportions: 90%–10% and 10%–90%; 95%–5% and 5%–95%; 97%–3% and 3%–97%. The samples were analyzed using both flow cytometry and Gel card kits.

**Table 1:** Volunteer phenotyping.

Volunteer	ABO Rh	C	c	E	e	Jka	Jkb	K	k	Fya	Fyb	S	s
1	A+	+	+	−	+	+	−	−	+	−	+	+	+
2	A+	+	+	+	+	+	−	−	+	+	+	+	−
3	A+	+	−	−	+	+	−	−	+	+	+	+	+
4	A+	+	+	−	+	+	+	−	+	−	+	+	+
5	A+	+	+	−	+	+	+	−	+	+	+	+	+
6	A+	+	+	−	+	+	−	−	+	+	−	−	+
7	A+	−	+	+	−	+	−	−	+	−	+	+	+
8	A+	+	−	−	+	+	−	−	+	+	+	+	+
9	A+	+	−	−	+	+	+	−	+	+	+	−	+
10	A-	−	+	−	+	+	+	−	+	−	+	+	+
11	A-	−	+	−	+	+	−	−	+	−	+	+	+
12	O+	−	+	+	+	−	+	−	+	+	+	−	+
13	O+	−	+	+	+	+	+	+	+	+	−	−	+
14	O+	+	+	+	+	+	+	−	+	+	−	−	+
15	O+	+	−	−	+	+	−	−	+	+	+	+	+
16	O+	+	+	−	+	+	−	−	+	+	−	−	+
17	O+	+	+	+	+	+	+	−	+	+	+	+	+
18	O+	+	+	+	+	+	+	−	+	−	+	+	+
19	O-	−	+	+	+	+	+	+	+	−	+	+	−
20	O-	−	+	−	+	−	+	−	+	−	+	+	+
21	AB+	+	−	−	+	+	+	−	+	+	+	+	+
22	B+	+	+	−	+	−	+	−	+	+	−	+	+

### Flow cytometry analysis

Phenotyping of volunteer blood samples and analysis of blood mixtures were performed as described by Mirotti et al. (22), with minor modifications. Briefly, 100 μl of homogenized whole blood was washed twice with Cell Stab buffer (BioRad, Diamed, Brazil), centrifuged at 1,500 g for 3 min between each wash step, and the resulting pellet was resuspended in Cell Stab buffer to a final volume of 1 ml. Cells were counted on a Sysmex XN-1,000 analyzer and diluted to 0.07 × 10^6^ RBC. A volume of 25 μl of cell suspension was added to 12 different wells of a 96-well round bottom polypropylene plate (Costar, Corning, USA) and washed with 175 μl of flow buffer (BSA 0.1%, Sodium Azide 0.05% in PBS). The final pellet was resuspended in 50 μl of different primary antibodies and incubated at room temperature for 30 min. The primary antibodies were against C, c, E, e, Jka, Jkb, K (IgM, Diaclon, BioRad, Switzerland), S, s, Fya, Fyb (IgG, ID-Antigen Profile III, BioRad, Switzerland) and k (IgG, Griffols Diagnostics AG, Switzerland). After two washes with 200 μl of flow buffer, the cells were incubated for 30 min protected from light, with 50 μl of the respective PE-conjugated secondary antibodies, anti-human IgM or IgG (Life Technologies, USA). Following three additional washes, the cells were resuspended in 200 μl of flow buffer, and sent to flow cytometry acquisition (FC-500, Beckman-Coulter, USA). Laser alignment was verified using Flow-Check Pro Fluorospheres beads (Beckman-Coulter, Ireland). A blood sample processed under the same conditions but without primary antibodies was used as negative control, while another blood sample incubated with the primary antibody CD235a-FITC (Beckman-Coulter, France) was used as positive control for RBC identification. Flow cytometry analyses were performed using CXP 2.2 software (Beckman-Coulter) and 50,000 single events were acquired.

### Gel card analysis

Blood mixtures were tested using the Gel card kit protocol as described in the manufactureŕs instructions. Briefly, all samples were centrifuged at 1,500 g for 3 minutes, and the plasma fraction was discarded. For the analysis of antigens C, c, E, e, and K, 25 μl of the concentrated RBCs were diluted in 0.5 ml of ID-diluent 2 (BioRad, Diamed, Brazil). Then, 12.5 μl of the resulting cell suspension were transferred to all microtubes of the ID-Card (Diaclon Rh-subgroups + K, BioRad, Diamed, Brazil). For the analysis of antigens k, Jka, and Jkb, 25 μl of concentrated RBCs were diluted in 0.5 ml of ID-diluent 1 (BioRad, Diamed, Brazil), and after 10 min of incubation at room temperature, 12.5 μl of the cell suspension were transferred to all microtubes of the ID-Card (ID-antigen Profile II, BioRad, Diamed, Brazil). For the analysis of S, s, Fya, Fyb antigens, 12.5 μl of concentrated RBCs were diluted in 1 ml of ID-diluent 2. Then, 50 μl of the resulting cell suspension were added to all microtubes of the ID-Card (ID-antigen Profile III, BioRad, Diamed, Brazil). Subsequently, 50 μl of the antibodies were added to their corresponding microtube and incubated for 10 min at room temperature. After incubation, all ID-cards were centrifuged for 10 min in an ID-Centrifuge 24 S (BioRad, Diamed, Brazil), and the results were analyzed using the Saxo ID-Reader (BioRad, Diamed, Brazil).

## Results and discussion

### Volunteer phenotyping

To select RBCs differing in the expression of 12 minor surface antigens (C, c, E, e, K, k, Jka, Jkb, Fya, Fyb, S, s), blood samples from 22 volunteers were phenotyped using the well-established cytofluorimetric method employed to detect HBT for doping control purposes (22) (Table 1). This assay involves incubating RBCs with specifically labeled fluorochrome antibodies targeting surface minor antigens, followed by analysis using flow cytometry. The peak on the right side of the histogram indicates that the volunteer's cells express the antigen, while the peak on the left side indicates the absence of expression. With regard to the major ABO and Rh RBC antigen systems, nine individuals were A+, two were A-, seven were O+, two were O-, one was AB+, and one was B+. All volunteers tested expressed k antigen, as expected, since the non-expression of this antigen is rare in the population (23).

### Blood mixture analysis

**Figure 1:**
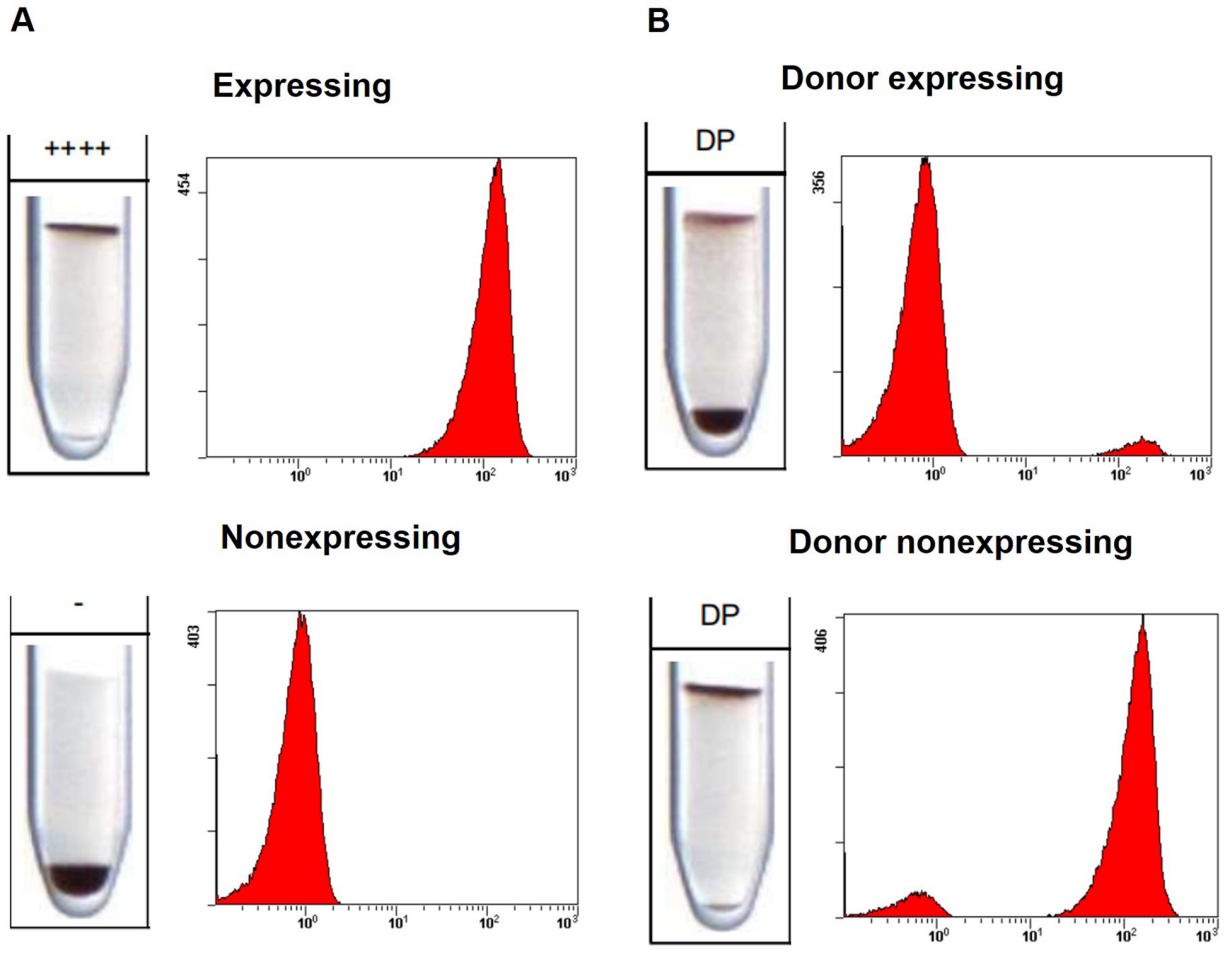
Comparison between Gel card and flow cytometry results. **(A)** Negative finding: single red blood cell (RBC) population, either expressing (RBCs located at the top of the Gel or a peak on the right side of the histogram) or not expressing (RBCs located at the bottom of the Gel or a peak on the left side of the histogram). **(B)** Adverse analytical finding: presence of a double RBC population either with donor cells expressing the antigen (minor population at the top of the Gel or a minor peak on the right side of the histogram) or double RBC population with donor cells non-expressing the antigen (minor population at the bottom of the Gel or a minor peak on the left side of the histogram). 10% of donor's cells were used. ++++ Strong expression detected,—no expression detected, DP double population detected.

Blood transfusion is used by athletes to increase red blood cell mass and enhance endurance performance. This method is prohibited by WADA, and doping control laboratories detect HBT using the robust and efficient cytofluorimetric method. Similar to the flow cytometry technique, the Gel card kit detects RBC populations that express or do not express a specific antigen. After Gel card centrifugation, cells expressing the targeted antigen interact with the specific antibody and concentrate at the upper part of the tube. Cells that do not express the antigen pass through the Gel, descend, and precipitate at the bottom. Similarly, after flow cytometry analysis, the peak on the right side of the histogram represents cells expressing the antigen, while the peak on the left represents cells that do not express the antigen, as shown in Figure 1A. The HBT event, a blood mixture containing RBCs expressing and not expressing the target antigen, exhibits a double population pattern in the Gel cards. Part of the cells will be located at the top, while the remainder will be placed at the bottom of the Gel (DP, double population), similar to the patter observed in flow cytometry, with two peaks in the histogram (Figure 1B).

**Figure 2:**
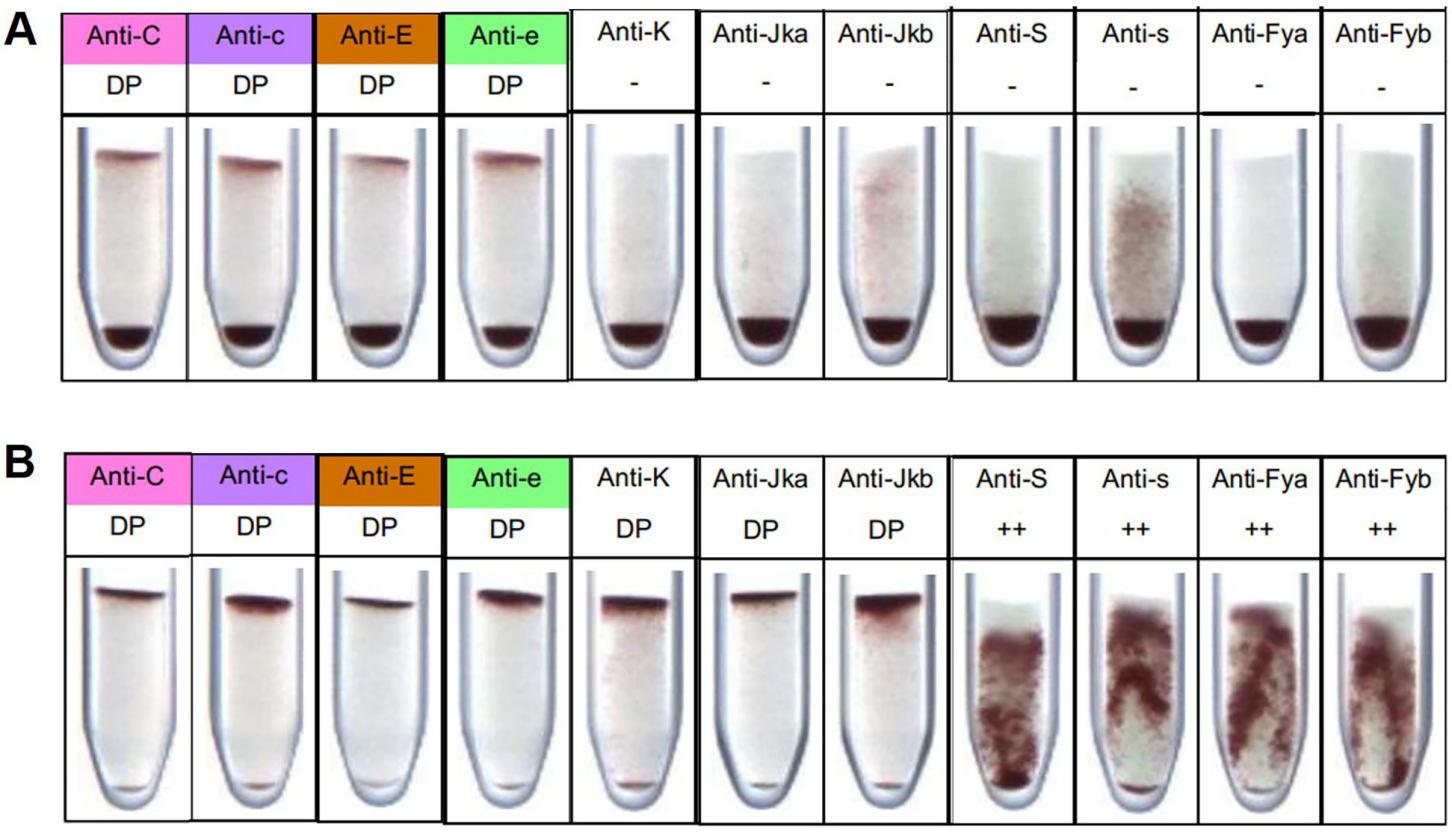
Detection of HBT events by Gel card, for each antigen tested. **(A)** Donor expressing the antigen. **(B)** Donor not expressing the antigen. 10% of donor's cells were used. ++ Weak expression detected,—no expression detected, DP double population detected.

To simulate an HBT event and test the capacity of Gel cards to detect double populations, blood from selected volunteers were mixed. Since Gel card technology is based on cell agglutination to detect the presence of an antigen, the blood mixtures were performed between compatible volunteers in terms of ABO system. For minor antigens, blood samples from non-compatible volunteers were mixed in a 10% donor blood to 90% recipient blood ratio. The ability of the Gel card to distinguish between two populations—those expressing and those not expressing C, c, E, and e antigens—was tested by mixing the whole blood from individuals 3 and 7. For the K antigen, blood from individuals 12 and 13 were mixed. To test for Jka and Jkb antigens, blood from individuals 12 and 15 were mixed. The absence of a volunteer who does not express the k antigen prevented testing for this antigen in the Gel card kits. To test Fya and Fyb, as well as S and s antigens, blood from individuals 4 and 6, and 2 and 6, respectively, were mixed. The Gel card successfully identified two RBC populations, those expressing and those not expressing the C, c, E, e, K, Jka, and Jkb antigens (Figure 2). However, this kit was unable to detect minor populations expressing the K, Jka, and Jkb antigens (Figure 2A), and only identified minor populations lacking expression of these antigens (Figure 2B). Detection of minor populations expressing the S, s, Fya, and Fyb antigens was also not observed (Figure 2A). In contrast to cells expressing C, c, E, e, K, Jka and Jkb antigens, RBCs expressing S, s, Fya and Fyb do not remain concentrated at the top of the tube after Gel card centrifugation, but are instead dispersed throughout the Gel. Therefore, this kit was unable to distinguish between cell populations of cells that express and do not express these antigens (Figure 2B). The inability of the Gel card to separate RBCs that express from those that do not express S, s, Fya, and Fyb was expected, due to the low expression of these antigens on the cell surface. As reported in the manufacturer's instructions, the presence of these antigens on the cell surface can still be confirmed even when RBCs are dispersed throughout the Gel, which corresponds to a weak but responsive reaction (++). However, to efficiently separate expressing cells from non-expressing cells, it is necessary that RBCs expressing the antigen remain at the top of the Gel.

**Figure 3:**
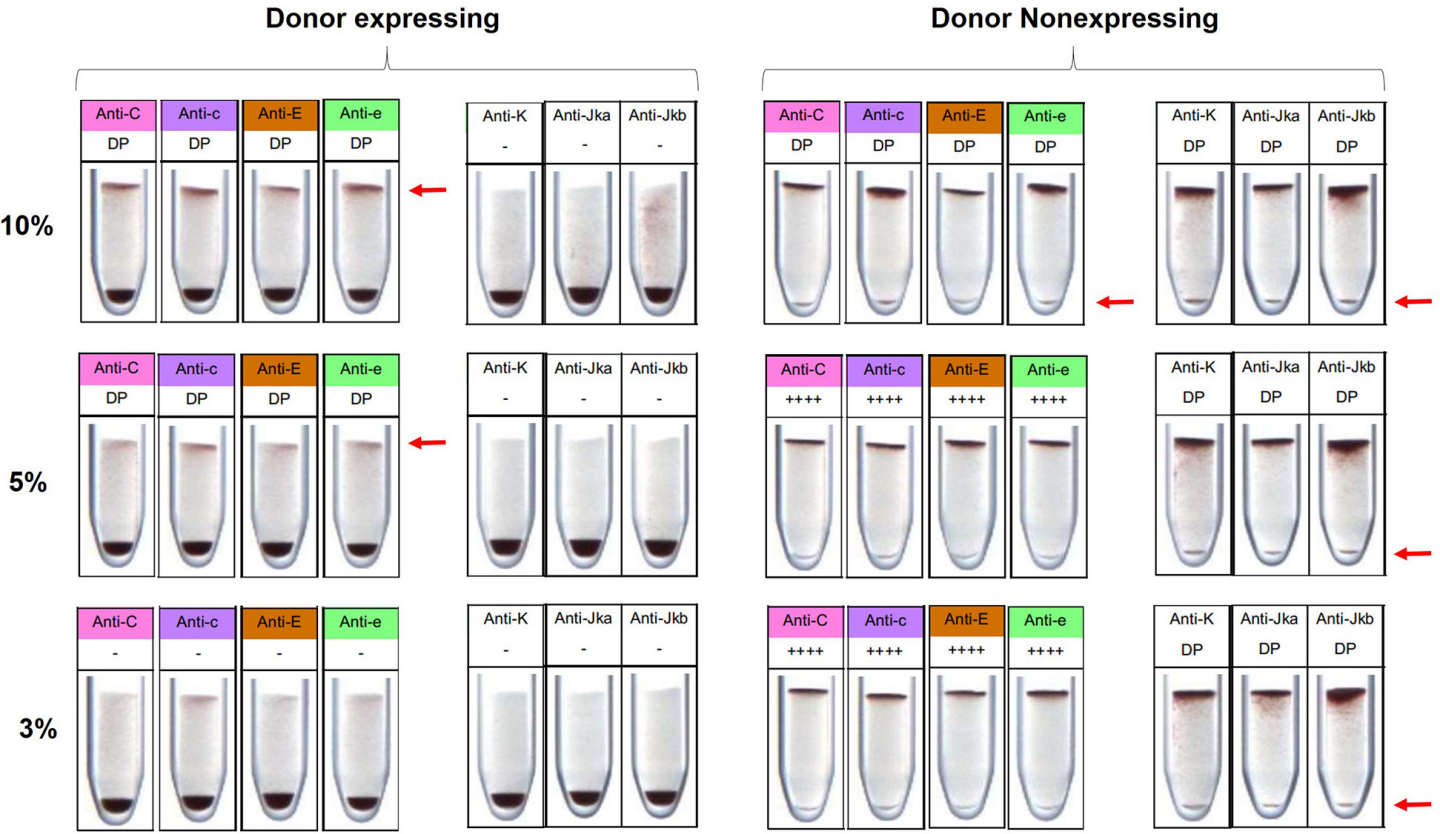
Limit of detection (LOD) for C, c, E, e, K, Jka and Jkb antigens by Gel card. For the C, c, E and e antigens, the limit of detection was 5% when the donor expressed the antigen, and 10% when the donor did not express it. For the K, Jka and Jkb antigens, donor cells were not detected when expressing the antigen, and were detectable at 3% when non-expressing. The red arrow indicates the detection of donor cells. ++++ Strong expression detected, — no expression detected, DP double population detected.

The cytofluorimetric method is capable of detecting less than 1% of donor cells in a blood mixture (10, 15, 16). To evaluate the LOD of this alternative technique, blood mixtures containing different proportions of donor cells (10%, 5%, and 3%) were tested using Gel card kits. The LOD varied depending on the antigen tested and whether the donor or recipient expressed the antigen. The LOD for the C, c, E, and e antigens was 5% when the minor population expressed the antigen, and 10% when the donor did not express it. For the K, Jka and Jkb antigens, the LOD was 3% when the minor population did not express the antigen. However, the kit was unable to detect double populations when the donor expressed these antigens (Figure 3).

Considering that HBT is a prohibited method outside of clinical transfusion protocols, the volume of blood used by athletes for doping purposes in such procedures can vary substantially, often based on informal reports. In a recent *in vivo* study, Marchand and co-workers identified several double populations of RBCs up to 50 days post-transfusion of 150 ml of RBC concentrate, using the flow cytometry protocol (11). It is clear that this traditional method is considerably sensitive for all tested antigens, and, consequently, the detection window is likely longer compared to the Gel card kit method. However, although microdosing enhance performance (4, 24), this effect depends on both the volume of transfused blood and the total blood volume of the athete, which varies between individuals. Considering an infusion of one or two whole blood bags (450 or 900 ml) (1, 25), such volumes could significantly increase hemoglobin concentration. In this context, the Gel card kits could be capable of detecting a recent HBT event, based on the LODs established in this study. Furthermore, Gel cards were not originally designed to separate RBC populations; however, the development of a device allowing the use of different antibody concentrations and/or cell numbers could enhance the ability to distinguish expressing from non-expressing cells, thereby lowering the LODs. Moreover, further studies are needed to evaluate the capability of Gel cards to separate RBC populations that express or do not express other antigens (e.g., M, N, P1, Le^a^, Le^b^, Lu^a^, Lu^b^), which would increase the antigen panel and reduce the risk of a false negative result. In addition, administration studies are essential to establish detection times post-transfusion and define the detection window. Despite the lower sensitivity of this technique compared to the flow cytometry method, it is important to consider that the Gel card method requires only 20 min for sample analysis, which is significantly faster than the approximately 5 h required by the traditional method. Another important aspect is the lower cost of analysis, estimated at around 30% of the cost of the cytofluorimetric method, when considering the same number of samples and antigens tested. Furthermore, the Gel card method does not require expensive equipment with routine maintenance,—such as a Flow Cytometer—or a highly specialized technician to operate it. This technique is simpler, faster, and more cost-effective, compared to the flow cytometry method, and it has the potential to be used as an ITP for anti-doping purposes—particularly in laboratories where flow cytometry equipment is not available—despite its reduced sensitivity and antigen coverage.

## Data Availability

The original contributions presented in the study are included in the article/Supplementary Material, further inquiries can be directed to the corresponding author.
